# Tilapia Skin in Burn Injuries: A Narrative Review of Pathophysiology, Current Management, and Therapeutic Applications

**DOI:** 10.7759/cureus.102556

**Published:** 2026-01-29

**Authors:** Mateusz Szot, Mikolaj Zakrys, Katarzyna Zakrys, Szymon Stupnicki, Aleksandra Oparcik, Jakub Tarczykowski, Natalia Kwasniewska, Mieszko Czaplinski

**Affiliations:** 1 General Practice, Uniwersytecki Szpital Kliniczny w Poznaniu, Poznań, POL; 2 General Practice, Provincial Hospital, Poznan, POL; 3 General Practice, Wojewódzki Szpital Wielospecjalistyczny im. Dr. Jana Jonstona w Lesznie, Leszno, POL; 4 General Practice, Centrum Medyczne HCP, Poznań, POL; 5 General Practice, Wojskowy Instytut Medyczny - Państwowy Instytut Badawczy, Warsaw, POL; 6 General Practice, Uniwersyteckie Centrum Klinicznym w Gdańsku, Gdańsk, POL

**Keywords:** better outcomes, burn wound management, cost-effectiveness of wound care, novel treatment, reconstructive plastic surgery, tilapia skin, wound healing and tissue repair

## Abstract

Burn wounds are common worldwide and often require prolonged treatment and recovery time. Such injuries are associated with local and systemic changes, both of which require effective action to prevent deterioration and improve long-term outcomes. The most common division of burns is based on injury depth. Treatment differs depending on the extent of the injury. The social and financial burden of burns is heavy, with some survivors being excluded from society. Due to high treatment costs and restricted availability of some management options, patients in low-resource settings may not receive the most advanced care. Therefore, new cost-effective and easily accessible management options are desired to improve clinical outcomes and provide an alternative to traditional methods. Nile tilapia skin showed promising results in burn wound treatment in preclinical and clinical studies. Unique antimicrobial, anti-inflammatory, and molecular properties of tilapia skin, combined with a structure similar to human cutis, are associated with enhanced wound healing and faster re-epithelialization. In animals, tilapia skin showed promising results in wound healing of various depths. In humans, superficial partial-thickness burns were mostly examined. In comparison to conservative management, patients treated with tilapia skin experienced lower pain intensity, required fewer dressing changes, and decreased analgesic intake. Re-epithelialization was faster. Advantages over traditional methods include a lack of local adverse reactions, low infection risk, reduced pain, cost-effectiveness, ease in tilapia skin acquisition, and application. Clinical studies restricted to a small number of participants and a lack of research on humans in various burn depths limit the broader employment of tilapia skin. Data on long-term outcomes are scarce; therefore, more studies need to be performed in that area to create clear guidelines and standardized treatment protocols. However, the beneficial effects of tilapia skin on wound healing are very promising and have high potential for development. In the future, it might become a cornerstone of burn injury treatment and improve clinical outcomes, particularly in low-resource settings.

## Introduction and background

Burns are traumatic injuries to the skin caused by heat, chemical compounds, electricity, friction, and radiation [1]. They represent the fourth most common type of trauma in general and the fifth most common cause of nonlethal injuries among the pediatric population [2,3]. Burns are an important issue worldwide, with the majority taking place in low- and middle-income countries [4]. The social burden of burns is heavy [4]. Despite intense treatment and prolonged recovery time, injury-associated disability and disfigurement are sometimes unavoidable [4]. Such patients may find it difficult to fit back into society due to altered external appearance and experience continued exclusion from social life [4].

Burn injuries are particularly challenging in low-income settings with restricted availability of advanced treatment options [5]. Therefore, less expensive alternatives are necessary, which are both clinically effective and easily available [5]. Tilapia skin can be considered as such [5].

Nile tilapia (*Oreochromis niloticus*) is a freshwater fish native to Africa and currently cultivated in Brazil and Thailand [5,6]. Its skin can be used in burn injury treatment as an alternative to traditional wound closure methods [6]. Tilapia skin features accountable for its wound healing properties include high collagen content, a structure similar to human skin, the capability to maintain proper moisture, and antimicrobial activity [6]. Patients may strongly benefit from the global application of tilapia skin in burn injury management [5,6]. Not only is it highly effective and demonstrates promising clinical outcomes, but it is also economical and widely available [5,6].

Methodology

This study is a narrative review of the recent literature on tilapia skin application in burn injuries. It also covers a brief explanation of burn pathophysiology, classification, and management. We performed a search of PubMed from inception through December 2025. For the introduction and the first part of the article, general burn-related terms were used (“burn overview”, “burn injury treatment”, “burns”, “pathophysiology of burns”, etc.). Mostly, recently published review articles were chosen, but some older papers were also included due to their thorough coverage of the topic. For the latter part of the review, keywords for tilapia skin and fish skin grafts (“fish skin graft”, “Nile tilapia”, “xenograft”, “tilapia skin”) were combined with burn-related terms (“burn injury”, “thermal injury”, “burns”). Recently published systematic reviews, in vitro, preclinical, and clinical trials were incorporated to present tilapia skin utility in the management of thermal injuries. As this is a narrative review, no statistical analyses were conducted. All statistical data and quantitative outcomes are based exclusively on the results of included studies.

Pathophysiology of burns

Burn injuries are associated with both local and systemic effects [7-9]. Locally, at the place of injury, three particular areas are differentiated: zone of coagulation - including irreversibly damaged necrotic tissue; zone of stasis - surrounding coagulation zone, containing damaged and ischemic tissue where inflammatory reactions take place and vasoconstricting factors are released; this area has potential to either heal spontaneously (in favorable conditions) or convert into full-thickness damage; zone of hyperaemia - the most external part of burn injury site, where blood vessels are dilated and perfusion is increased; and the risk of necrosis is very low unless there is dysfunction of blood supply to the region caused by septic shock or prolonged hypoperfusion [7-9]. Systemic consequences of burns involve cardiovascular, respiratory, immune, and other body systems [1,10]. In the cardiovascular system, reduced cardiac output, decreased cardiac muscle contractibility, and constriction of peripheral blood vessels might occur [10,11]. After hours or days of such cardiac depression, the body enters a hyperdynamic state where heart rate may be maintained at an elevated level for up to one year [10,11]. Prothrombotic state, impaired fibrinolysis, hyperinflammation, immune compromise, hypermetabolism (that might be present for years after injury), muscle wasting, and gut barrier dysfunction are also observed [10,12]. New therapies targeted at systemic changes encompass improving tissue perfusion, decreasing microvascular leakage, and coupling between the central nervous system and cardiovascular system [10,13]. They are believed to improve outcomes for patients with burn injuries [10,13].

Classification of burns

Depth is the most significant factor in burns classification [4,8,14]. According to this criterion, three main groups of burns can be differentiated: superficial (first degree, moderately painful), partial thickness (second degree, painful), and full thickness (third degree, minimally painful) [4,8,14]. Superficial burns involve only the epidermic layer and usually heal within 5-10 days without scars [4,8,14]. Partial-thickness burns can be further divided into superficial partial-thickness burns (SPTB - affecting the superficial, papillary layer of the dermis) - healing within two to three weeks with little scarring, and deep partial-thickness burns (DPTB - reaching deeper, reticular dermis) - in some cases possible to heal without surgery, however, with inevitable scarring [4,8,14]. Full-thickness burns (FTB) extend even further and affect not only epidermis and dermis but also subcutaneous tissue [4,8,14]. The healing process requires more than eight weeks, and surgical management is necessary [4,8,14]. This type of burn is painless; in such deep injuries, nerves are damaged [4,8,14]. Fourth-degree burns surpass subcutaneous tissue and reach more deeply located structures such as bones, tendons, and muscles [13].

## Review

Overview of burn injury treatment

Immediately after burn injury, healthcare providers ought to address priorities essential for survival, which are airways, breathing, and circulation [15]. Depending on the circumstances, additional measures might be necessary, such as endotracheal intubation to maintain airflow through obstructed airways, 100% oxygen flow, and a ventilator to support breathing [15]. Optimal fluid resuscitation is crucial to prevent hypoperfusion; however, it is worth noticing that excessive fluid supply might lead to complications, including pulmonary edema, heart failure, and compartment syndromes [15]. A Foley catheter should be inserted to monitor urine output and adjust fluid resuscitation accordingly [15]. Escharotomies and fasciotomies might be necessary to prevent tissue ischemia [15].

Early nutritional support and pain management are necessary to battle the hypermetabolic state [13]. Propranolol, adrenergic receptor blockers, and anabolic agents such as growth hormone, insulin, and oxandrolone (testosterone analog) might reduce post-burn catabolism [13]. Physical exertion and properly selected exercises prevent muscle loss and improve outcomes [13]. All burn wounds should be cleaned at presentation to minimize infection risk [13]. Systemic antibiotics should be administered only in infected wounds [13]. Antibiotic prophylaxis ought not to be used as it enhances the risk of multiple drug-resistant organisms formation [13]. Constant monitoring for sepsis and early treatment if necessary are required to prevent multiorgan failure and death [13].

Superficial burns heal spontaneously and do not require advanced treatment. Partial-thickness burns healing strongly depends on epidermal basal cells [13]. They can migrate 1-2 cm from the wound edge or up and across from dermal adnexa [13]. Contraction might appear in deep wounds deprived of appendages [13]. Proper wound moisture promotes optimal re-epithelialization and healing and reduces scarring [6,13]. Wound dressings made of natural or synthetic polymers are commonly used [16]. Closed, long-term, biologic dressings are the most preferred and include plant or animal products [13]. Xenograft skin coming from fish (including Nile tilapia) or mammals is in use [17]. Ointment can be used to maintain a moist environment [13]. Silver sulfadiazine (SS) 1%, an antibacterial cream, is widely used in the treatment of burns and positively impacts re-epithelialization [17]. Surgical treatment comprises early debridement, grafting, and reconstruction [18]. Early and proper debridement is highly important in the case of deep partial-thickness and full-thickness wounds [18]. Enzymes or hydrodissection may be used as an alternative to traditional surgical debridement [18]. The only possible healing processes in FTB are either contraction or skin grafting [13]. Available options include full-thickness or split-thickness skin grafts [13]. Currently, autologous skin grafting (autografts) preceded by surgical debridement is considered to be the gold standard for deep burn treatment [4,16,18]. Massive burns pose a challenge of limited donor sites [5,13]. In such cases, temporary skin substitutes or alternative wound closure methods are used, or grafting is delayed until donor sites heal and re-harvesting becomes possible [5,13,19]. However, these methods carry the risk of numerous complications, including pain, scarring, and infections [4]. Therefore, it remains necessary to continue the search for better, more effective, affordable, and scar-free healing skin substitutes [4]. Biological skin substitutes include materials coming from the same species (allografts) or from different species (xenografts) [16].

Wound healing properties of tilapia skin

Tilapia skin features unique properties stimulating the wound healing process [20-24]. It has been proven that Nile tilapia skin peptides play a significant role in the modulation of molecular and cellular mechanisms in animal and in vitro models [20-24]. They demonstrate antioxidant, antimicrobial, and anti-inflammatory features and increase both epithelial cell proliferation and migration in vitro [20-24]. Tilapia skin antimicrobial and anti-inflammatory properties (even against Methicillin-resistant *Staphylococcus aureus* - MRSA) are at least partially associated with its lipid profile, rich in omega-3 polyunsaturated fatty acids with large concentrations of docosahexaenoic acid (DHA) and eicosapentaenoic acid (EPA) [17,25].

Additionally, compounds and particles contained in tilapia skin stimulate angiogenesis, increase the formation of new blood vessels, promote granulation tissue formation, and support the synthesis of collagen fibers [20-24]. Cell adhesion, proliferation, and differentiation are improved [6]. Nile tilapia skin is structurally similar to human skin and, therefore, is characterized by high biocompatibility with human tissue and serves very well as a potential wound dressing [22]. It is mainly composed of type I collagen, which provides structural support and is vital in both tissue regeneration and extracellular matrix formation [6,20].

Tilapia skin dressings play an additional role as a barrier between the bed wound and external environment, preventing microbial contamination [6]. Additionally, tilapia skin shows good adherence to the wound bed owing to a porous structure with large diameter apertures allowing the passage of human fibroblasts [25,26].

Preclinical studies of tilapia skin application in wound treatment

Studies showed that collagen isolated from tilapia fish skin, applied in the form of nanoparticle gels on full-thickness open wounds in rabbits, promoted effective wound healing and presented excellent moisture retention capabilities [27]. Quicker and better re-epithelialization was observed along with increased cellular proliferation and collagen production at the wound site [27]. Exudation, inflammation, and microbial contamination were significantly decreased in comparison to wounds treated with saline only [27]. The levels of inflammatory cytokines, such as tumor necrosis factor-alpha (TNF-α) and interleukin 6 (IL-6), are significantly decreased in burn models treated with tilapia skin [28]. Another study examined how collagen extract obtained from Nile tilapia skin affects full-thickness wound healing in rats [29]. The results revealed that the test group treated with tilapia collagen expressed higher levels of angiogenic factors and promoted cutaneous wound healing [29]. It occurred by enhanced angiogenesis, production of chemotactic growth factors, and fibroblast proliferation [29]. In scalded white rabbits, fish skin peptides accelerated wound healing time in deep partial-thickness wounds [23]. Additionally, tilapia skin showed anti-nociceptive properties [30]. In full-thickness skin injuries in dogs and donkeys, tilapia skin dressing accelerated epithelialization and promoted wound healing [31,32].

Preclinical studies of tilapia skin employment in burn wound treatment

The study conducted by Garrity et al. compared the efficacy of burn wound healing between two groups of mice with FTB [4]. The study group was treated with tilapia skin, and the control group with a hydrocolloid adhesive bandage (HDC) [4]. Mice treated with tilapia skin exhibited higher antimicrobial peptide expression and enhanced angiogenesis at the earlier time point than HDC-treated controls [4]. No differences in wound closure time were detected between the two groups [4]. However, authors underscored the fact that the absence of wound closure acceleration in mice treated with tilapia skin might have resulted from differences in experimental design and animal species [4]. The mechanism of the wound healing process in mice is based on contraction of the dermis-associated panniculus carnosus muscle [4]. It is different from that in donkeys and humans, where formation of granulation tissue is key, followed by re-epithelialization [4]. This could explain the absence of accelerated wound closure, which, on the contrary, was observed in other studies conducted on different animals such as donkeys [4,33]. Authors emphasized the necessity to measure tilapia skin effectiveness in larger animal models and create different study designs to appropriately evaluate the wound closure effect [4]. A different study on rats with DPTB assessed a novel hydrogel wound dressing with collagen extracted from tilapia skin [34]. Wound healing properties of collagen dressings were compared with the positive control group treated with a hydrocolloid thin dressing and the blank control group [34]. The results revealed that tilapia skin collagen hydrogel dressing significantly improved the healing of wounds, increased the maturation of skin adnexa, and formation of epidermal layers [34].

Idea of tilapia skin application in burn injuries in humans

The first description of Nile tilapia fish skin application in burn injuries was published in 2019 by a Brazilian team led by Lima Junior [26]. Back then, available literature revealed that tilapia skin was colonized only by non-infectious microbiota, contained collagen of a structure similar to human skin, and was safe to sterilize without altering its microscopic or tensiometric characteristics [35,36]. Additionally, it showed promising results in burn treatment in rats [26]. Based on the provided literature, the authors sterilized and irradiated the skin before application [26]. The patient was 23 years old and suffered SPTB on the right upper limb and DPTB on the left upper limb, face, and thorax when he came in contact with flames from a gunpowder explosion [26]. Additionally, 16% of the total body surface area (TBSA) was involved [26]. The wound was cleaned, and a tilapia skin dressing was placed on the wounds of the upper extremities [26]. After 12 and 17 days, respectively, the dressings were taken off, and full re-epithelialization was present. It confirmed that tilapia skin can be effectively used in the management of at least some types of burn injuries [26].

Clinical outcomes of tilapia skin application in burn injuries

Lima Junior et al. conducted four clinical trials researching tilapia skin xenograft effectiveness in the management of SPTB wounds [37-40]. Three of them compared tilapia skin to 1% SS, whereas one compared carboxymethylcellulose dressing with 1.2% silver (naCMG-Ag) [37-40]. All studies revealed that patients treated with xenografts required fewer dressing changes, and the differences were statistically important in all of the studies [37-40]. Three out of four papers confirmed reduced intake of some analgesic drugs during anesthetic procedures in patients treated with tilapia skin [37-40]. Furthermore, three of the studies compared re-epithelialization time between research and control groups [37,38,40]. All proved that the process was faster in patients treated with xenografts [37,38,40]. For example, in adult outpatients with SPTB involving less than 10% of TBSA, time to complete re-epithelialization totaled 9.77 ± 0.83 days with tilapia skin vs 11.20 ± 0.63 days with SS (p=0.0002) [38]. Inpatients with SPTB involving 10%-20% of TBSA also exhibited faster time to full re-epithelialization - 10.56 ± 1.12 days with tilapia skin vs 11.70 ± 0.67 days with SS (p=0.0147) [38]. Study on pediatric patients with SPTB involving less than 20% of TBSA showed faster re-epithelialization (10.07 ± 0.46 days with tilapia skin vs 10.47 ± 0.74 days with SS), although the result was not statistically significant (p=0.0868) [37].

One study conducted by Lima Junior et al. incorporated not only patients with SPTB but also DPTB [38]. In this group, patients treated with xenografts also showed faster re-epithelialization - 18.10 ± 0.99 days with tilapia skin vs 21.30 ± 1.42 days with SS (p=0.0001) [38]. Patients treated with tilapia skin experienced lower pain intensity and required decreased analgesic intake during anesthetic procedures (ketamine, dipyrone, fentanyl) [38]. The number of necessary dressing changes through treatment was also reduced - 6.10 ± 2.02 changes with tilapia skin vs 20.20 ± 1.69 changes with SS (p<0.0001) [38].

Advantages of tilapia skin application vs other treatment options

Tilapia skin dressing demonstrates numerous advantages over traditional methods of treatment in burn and normal wounds. In comparison to collagen alginate or SS cream, tilapia skin dressing requires less frequent changes, which are painless and associated with reduced need for analgesic drugs [6,25,26]. Due to structural similarities between tilapia and human skin, no adverse reactions are observed in patients treated for chronic wounds, which, on the contrary, might happen with traditional dressings [25]. In comparison to autografts, tilapia skin dressing eliminates the risk of donor site morbidities [25]. There is also no risk of rejection, which is an existing issue in allografts [25].

From an economic perspective, tilapia skin dressing seems to be cheaper than the currently used alternatives [40]. There is one study that directly compared the costs of tilapia skin and 1% SS cream in SPTB treatment [40]. In tilapia skin-treated patients, the mean costs for each burned 1% of TBSA were reduced by approximately 50% when compared to SS cream [40]. These promising results demonstrate that tilapia skin is more economical than some traditional treatment options and can be used as an alternative, particularly in low-income areas [40]. The cost-effectiveness is even more significant when the tilapia skin price is juxtaposed with more advanced synthetic skin substitutes [6].

Finally, tilapia skin can be easily obtained from in-house aquaculture or as a secondary product from fish farms [4-6,25]. It is a major advantage over synthetic dressings, allografts, and autografts with restricted accessibility due to product insufficiency or limited availability of donor sites [4-6,25]. Worldwide application of tilapia skin in burn wound treatment may not only become a very cost-effective solution and provide substantial benefits for patients in general but also significantly improve outcomes and availability of quality treatment in resource-limited healthcare settings [4-6,25].

Tilapia skin vs other xenografts

In terms of mammalian xenografts, porcine, bovine, and ovine acellular dermal matrices are used in the treatment of burns [19,41]. They increase skin re-epithelialization, cell proliferation, and support wound healing [41]. However, some beneficial effects of graft-included biocomponents, such as lipids, glycans, elastin, and hyaluronic acid, are mitigated by detergents and viral inactivation measures [41]. These processes are necessary to reduce infection transmission risk and provide safety for graft recipients [41]. Secondly, porcine grafts cannot be applied to some patients due to religious reasons [41]. Additionally, mammalian grafts exhibited longer re-epithelialization and contraction time than fish skin grafts (FSGs) [41,42].

Therefore, FSGs seem to be a better alternative [41]. They have no documented risk of zoonotic transmission and require a less intense preparation to assure recipient safety [41]. Reduced processing is less expensive and ensures preservation of pro-healing biocomponents, such as proteoglycans, soluble collagen, elastin, lanolin, fibronectin, and omega-3 polyunsaturated fatty acids - EPA and DHA [16,25,41]. Particularly, the latter ones proved their antimicrobial, anti-inflammatory, and anti-nociceptive properties [41]. Additionally, FSG application on humans does not pose any religious concerns, which is an existing issue with porcine grafts [41].

To date, four FSG species are available: North Atlantic cod (commercially available as Kerecis®), Nile tilapia, silver carp, and grass carp. Data on grass and silver carp FSGs are limited as these two species were studied only in preclinical studies on animal models [41]. Therefore, Atlantic cod and Nile tilapia are the only FSGs with published clinical trials assessing their application and clinical outcomes in burns [41]. FSGs coming from these species share common features. Both are biocompatible with human skin, effective in burn wound management, and exhibit antimicrobial, anti-inflammatory, and pro-healing properties [5,25,41,43].

Similar to Nile tilapia, grafts coming from North Atlantic cod require minimal processing before application and pose a low risk of viral or prion disease transmission [41,43]. The Kerecis® product proved to induce early granulation sooner and allowed for a step-down in the reconstruction ladder in austere, war-conflict surroundings [43]. Minimal training is required for medical personnel, while application, storage, and transportation remain easy and non-demanding [43].

There are no studies directly comparing the efficacy or cost-effectiveness between Nile tilapia and North Atlantic cod FSGs [41]. However, in many studies, it was underscored that application of tilapia skin in burn injuries might be a cost-effective alternative to standard management and improve outcomes, particularly in low-resource healthcare settings [6,37-40].

Current limitations in the broader application of tilapia skin 

Many studies concerning tilapia skin application in burn treatment were conducted on small groups of patients, and further testing in larger cohorts is required [25]. To incorporate tilapia skin more broadly, clear guidelines ought to be created on how to sterilize (this process may alter the properties of tilapia skin), prepare, and apply the xenografts to provide the most effective treatment [25]. Meanwhile, it is vital to preserve its healing properties and low infection risk [4,5]. Studies differ in preparation methodology (i.e., irradiation, freezing, microbial testing techniques), dressing time changes, and form of applied tilapia skin - either as a xenograft, hydrogel dressing, or bioactive extract [4,5]. Additionally, in the case of xenografts, some of them are lyophilized, while others are glycerolized [4,5]. It poses difficulties in comparison and interpretation between the studies [4,5].

Furthermore, current data on Nile tilapia skin application in human burn wounds is limited to a few case reports and clinical trials restricted mostly to SPTB [6,37-40]. Only one study conducted on humans involved DPTB wounds [38]. No randomized clinical trial on tilapia skin application for FTB is currently available [41]. There is a lack of research assessing long-term outcomes such as scarring, functional recovery, patient satisfaction, and quality of life [5]. In terms of aesthetics, some patients and medical professionals may be biased against fish skin application in wound treatment due to the altered appearance of the skin during treatment [5]. Finally, if tilapia skin were to become more commonly used, supply chains and distribution networks need to be established to meet the demand [5].

## Conclusions

Dealing with patients suffering from burns requires complex management. Addressing priorities essential for survival (airways, breathing, circulation, systemic effects) and wound care is essential to achieve good clinical outcomes. Wound treatment itself is associated with numerous complications and challenges; therefore, it is vital to search for better, more effective, and affordable treatment methods. Tilapia skin proved its wound healing properties in preclinical and clinical studies. Faster re-epithelialization and activity against pathogens are only some of the tilapia skin qualities that place it among modern advances in burn wound management. Studies confirmed the use of tilapia skin in burn injuries. In humans, particular advantages are observed in SPTB and DPTB wounds. Promising results were shown in the treatment of full-thickness skin wounds in animal models, but no clinical trials concerning FTB management are available, which limits tilapia skin deployment in a broader context.

Large, multicenter trials on diversified populations are recommended to fully examine and validate the effectiveness of tilapia skin in various clinical settings and different burn depths. Additionally, the long-term effects of tilapia skin treatment need to be assessed to obtain full information. Consequently, the establishment of evidence-based tilapia skin treatment protocols would bring the best clinical outcomes. In the future, tilapia skin may become a standard in burn wound treatment. This novelty might be particularly beneficial in low-resource settings due to its affordability and accessibility. Still, more health-economic analyses are needed to fully research the cost-effectiveness of tilapia skin application in burns. Combining tilapia skin with stem cell treatment or application of FSGs in various types of wounds may be another interesting direction of tilapia skin research and development. Exploration of fish skin application in military medical facilities and field hospitals might also improve outcomes and benefit patients.
